# Sero-Occurrence of HBV/HCV Co-infection and Levels of Liver Enzymes among Patients at a Tertiary Care Hospital in Central India: a Pilot Study

**DOI:** 10.5195/cajgh.2019.313

**Published:** 2019-03-29

**Authors:** Prabha Desikan, Aseem Rangnekar, Zeba Khan, Nikita Panwalkar, Protiti Bose, Hanni Vasudev Gulwani, Sukhpreet Kaur

**Affiliations:** 1 Department of Microbiology, Bhopal Memorial Hospital and Research Centre, India; 2 Department of Pathology, Bhopal Memorial Hospital and Research Centre, India

**Keywords:** Hepatitis B, Hepatitis C, Co-infection, India

## Abstract

**Introduction:**

Hepatitis B and C viral infections share common modes of transmission and account for a large proportion of liver disease burden across the globe. Patients with Hepatitis B (HBV) and Hepatitis C virus (HCV) co-infection may have more severe liver disease and are potentially at higher risk for developing hepatocellular carcinoma. The aim of this study was to assess the sero-occurrence of HBV/HCV co-infection by examining the medical records of tertiary care hospital patients in Central India and determine the extent of liver damage based on liver function tests (LFTs).

**Methods:**

Patients with a positive test for HBV surface antigen (HBsAg) over a period of 10 years were identified from laboratory records in a tertiary care facility in central India. Records of 51,075 consecutive non-duplicate blood samples were then screened for a positive HBV and HCV tests. LFT, liver enzymes, and bilirubin data were also extracted. Means and standard deviations were determined for continuous variables, and the difference in means was compared using a independent samples t-test. Associations between HBV/HCV co-infection status and demographic variables were calculated using Pearson’s Chi-squared test. A p-value less than 0.05 was considered statistically significant.

**Results:**

In this study, 1674 (3.27%) screened patients were positive for HBsAg and the sero-occurrence of co-infection with HCV in HBsAg positive patients was reported in 28 individuals (1.67%). There was no significant gender difference for HBV/HCV co-infection (p>0.05). HBV/HCV co-infection was observed more frequently in the 31–60 year old age group (p=0.001). HBV/HCV co-infected patients had significantly higher levels of liver enzymes and bilirubin than those with HBsAg mono-infection (p=0.001).

**Conclusion:**

Liver function tests are potentially important predictors for HBV/HCV coinfection. Screening for HCV co-infection in HBsAg-positive patients is recommended in India. Detection of co-infection may enable timely preventive/therapeutic interventions aimed at preventing progression to hepatocellular carcinoma.

## Introduction

Chronic liver disease (CLD) results from an inflammatory injury to the liver, persisting for six or more months without complete resolution. CLD can result from a spectrum of diseases including chronic hepatitis, liver cirrhosis, and hepatocellular carcinoma (HCC)^1^. Chronic Hepatitis B virus (HBV) and Hepatitis C virus (HCV) infections are significant public health issues globally. Compared to HBV mono-infected patients, HBV/HCV co-infected patients have higher rates of cirrhosis (44% vs. 21%) and decompensated liver disease (24% vs. 6%). Similarly, HBV/HCV co-infected patients compared to HCV mono-infected patients, have a higher rate of cirrhosis (95% vs. 49%) and more advanced decompensated liver disease (Child-Pugh class C 37% vs. 0%)^2^. Epidemiologic studies in patients with dual HBV/HCV infection demonstrated an increased risk of HCC, which has been confirmed by three published meta-analyses^3^–^5^. Evidence suggests that HBV is capable of initiating the neoplastic process, while HCV can act as a promoter, and that the two conditions may be synergistic in leading to HCC development^3^.

The estimated global prevalence of HBV/HCV co-infection is approximately 5–20% in HBsAg positive patients and 2–10% in HCV positive patients^6^. In India, the prevalence of HBV and HCV co-infection ranges from 1.89% to 56% depending on the region^7^,^8^, which may be related to a lack of awareness of HBV vaccine availability by the general public^9^. HBV/HCV co-infection is more frequent in high-risk populations of various ages, including intravenous drug users, patients receiving hemodialysis, patients undergoing organ transplantation, HIV-positive patients, and β-thalassemia patients^10^. Previous studies reported that age below 40, Asian race, injection drug use, a greater number of lifetime sexual partners, inadequate sterilization of medical equipment, unprotected sex, and the use of unscreened blood and blood products are independent risk factors for HBV/HCV dual infection^10^,^11^. In India, the most commonly reported modes of transmission of HBV and HCV are sexual transmission, blood transfusion, and intravenous drug use^12^. Moreover, because of the shared modes of transmission, HBV/HCV co-infections are common in endemic areas and among subjects with a high risk of parenteral transmission^13^,^14^ Study findings report a greater likelihood of the progression from chronic hepatitis to cirrhosis and HCC in patients with HBV/HCV co-infection compared to infection by HBV or HCV alone; this combination of cirrhosis and HCC is particularly difficult to clinically manage^15^.

Measurement of liver enzymes function (i.e. Alanine transaminase (ALT), Aspartate transaminase (AST), and Alkaline Phosphatase (ALP)) is an inexpensive and non-invasive method of assessing liver disease. Measurement of liver enzymes reflect the activity of hepatotropic viruses and the degree of damage to the liver during therapy with various hepatotoxic drugs^16^. To our knowledge, very limited data about the sero-occurrence of HBV/HCV co-infection are available from Central India. The objectives of this study are to (1) identify the prevalence of sero-occurrence of HBV/HCV co-infection in a large tertiary care facility, and (2) determine the extent of liver damage in patients with HBV/HCV co-infection.

## Methods

### Study design and data collection

The study was carried out in the Department of Microbiology, Bhopal Memorial Hospital & Research Centre (BMHRC), Bhopal, and Madhya Pradesh, India. BMHRC is a tertiary care hospital and serves a population of more than two million residents. Records of patients and test results were maintained in lab registers as well as a hospital information system (HIS) of the BMHRC. Laboratory records from January 2006 to December 2016 were reviewed retrospectively for demographic and HBsAg data. Lab records were also reviewed for anti-HCV sero-positivity among HBsAg positive samples. This study was limited to samples from patients who visited either outpatient (OPD) or inpatient departments (IPD) of BMHRC, and who were diagnosed as HBsAg and anti-HCV positive. Samples with incomplete liver function panel or demographic data were excluded. All the investigations for HBsAg and anti-HCV were completed by Enzyme Linked Immunosorbent Assay (ELISA) as part of routine laboratory work flow, in accordance with the manufacturer’s instructions. Liver enzyme tests were done by biochemical assays, and included Alanine transaminase (ALT), Aspartate transaminase (AST), Alkaline Phosphatase (ALP), bilirubin total (BT), bilirubin indirect (BI), and bilirubin direct (BD). The study was approved by the Institutional Ethical Committee of BMHRC.

### Statistical analysis

Data were analyzed using OpenEpi online software^17^. Differences in variable distribution were evaluated by Pearson’s chi-squared test, and a p-value of less than 0.05 was considered statistically significant. Means and standard deviations were reported for continuous variables, and differences in means were compared with an independent samples t-test. We defined abnormal liver enzyme cut-offs according to the National Health and Nutrition Examination Survey (NHANES) criteria III^18^. Abnormal values of liver enzymes and bilirubin levels were defined as ALT/AST≥50 IU/ml, ALP≥129 IU/ml.BT/BI ≥1.1 mg/dl, and BD≥ 0.3 mg/dl.

## Results

**Figure 1:**
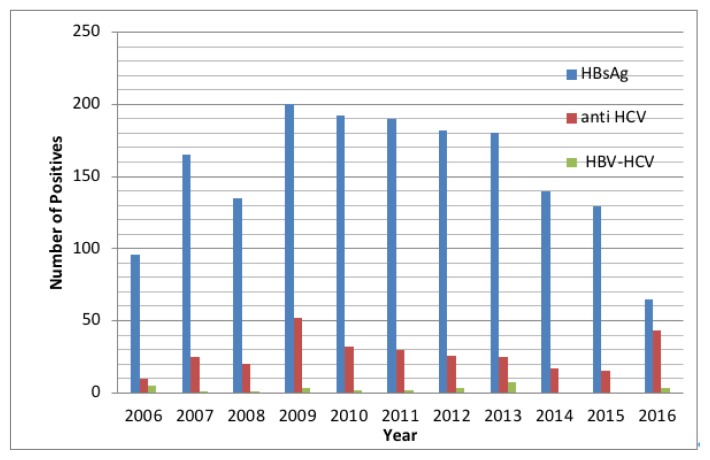
Trends of sero-positivity of HBsAg, anti-HCV, and HBV/HCV co-infection at Bhopal Memorial Hospital and Research Center in Central India 2006 – 2016.

A total of 51,075 consecutive non-duplicate blood samples were collected over a period of ten years for HBsAg testing. Out of these samples, 1674 (3.27%) were positive for HBsAg, and 295 (0.57%) were found to be positive for anti-HCV. Among individuals positive for HBsAg or anti-HCV, 28 (1.67%) samples were identified as positive for both, HBsAg and anti-HCV. Sero-occurrence rates are summarized in Table 1. Ages of patients positive for HBV ranged from 12–75 years of age (Median=54 years, IQR: 15), and 29–74 years of age (Median=45 years, IQR: 26) for HBV/HCV co-infected individuals. HBV/HCV co-infection was more frequent in males at 78.5% (n=22) than in females at 21.4% (n=6), but the difference was not statistically significant (p=0.80). A significantly higher prevalence of HBV/HCV co-infection was observed in the 31–60 years (46.4%) age group compared to the 1–30 years (25.0%) and >60 years (28.6%) age groups (p=0.001) (Table 1). The proportion of sero-occurrence of HBV/HCV co-infection fluctuates between 2006 and 2016 with the highest prevalence in 2013 (Figure 1).

Out of 1674 HBsAg positive samples, LFT results were available for 773 samples. The mean values of serum levels of Alanine transaminase (ALT), Aspartate transaminase (AST), Alkaline Phosphatase (ALP), bilirubin total (BT), bilirubin indirect (BI), and bilirubin direct (BD) were 114.4±403.8 IU/L, 94.7±344.8 IU/L, 101.4±71.6 IU/ml, 1.4±2.9mg/dl, 0.7±2.7mg/dl, and 0.7±1.7mg/dl respectively in HBsAg positive patients, and 100.25±201.4 IU/L, 106.3±265.7 IU/L, 114.7±65.9 IU/ml, 1.7±2.7mg/dl, 1.05±2.7mg/dl, and 0.7±0.7mg/dl respectively in HBV/HCV co-infected patients. Levels of ALT, AST, ALP, and bilirubin were significantly different between HBV/HCV co-infected patients and HBsAg mono-infected patients (p <0.001) (Table 2).

## Discussion

In this study, sero-occurrence of HCV co-infection in HBV patients was found to be low (1.67%) compared to global data, but higher than reported in other parts of India^7^,^12^,^19^. A large multicenter study from the United States assessed the prevalence of HBV/HCV co-infection and found anti-HCV was present in 7% of chronic HBV carriers^10^. Tesfa et. al. reported a 6.39% prevalence of HBV/HCV co-infection in a hospital-based study conducted in Ethiopia^20^. Studies conducted in different regions of India have reported diverse prevalence rates of HBV/HCV co-infection in drug abusers having chronic liver disease (1.5 %), patients on hemodialysis (0.8%), and in patients with HIV infection (3.0%)^7^,^21^–^22^.

While not statistically significant, we found that the seropositivity rate of HBV/HCV co-infection among males was higher than in females. This is an interesting finding, as it corroborates a previous report that male subjects are at a higher risk of developing HBV/HCV co-infection compared to females^23^–^24^. The reason for a higher frequency of HBV/HCV co-infection among male patients could be a result of a higher level of exposure to risk factors associated with co-infection, including drug abuse, unprotected sex, and having more than one sexual partner^25^.

Our study showed that HBV/HCV co-infection rates were highest among individuals in the age group of 31–60 (46.4%), followed by the age group of 60 years and over (28.57%), which was similar to a previously published study in the United States^10^. A higher prevalence in the 31–60 years age group may be associated with increased exposure to risk factors for HBV/HCV co-infection.

HBV/HCV co-infected patients in this study had significantly higher levels of ALT, AST, and ALP compared to HBsAg and anti-HCV mono-infected patients. Higher levels of liver enzymes have been reported in HBV/HCV co-infected patients compared to those having HBV and HCV mono-infection^26^. Bilirubin concentrations were found to be highest in co-infected patients in this study. A study conducted in tribal populations of Central India also found an elevated level of bilirubin in HBV/HCV co-infected patients^27^–^28^. It is documented that elevated levels of liver enzymes in HBV infected patients are associated with higher risk of liver cirrhosis and HCC^29^. Once HBV infection is detected during a diagnostic workup of hepatitis, it is possible that hepatitis may be attributed to HBV infection alone and further etiological diagnoses may not be carried out. This may lead to underreporting of co-infection with HCV. Therefore, detection of co-infection may enable timely intervention to prevent progression to HCC.

This pilot investigation is the first study to show sero-occurrence of HBV/HCV coinfection within the central zone of India, along with the comparison of liver enzymes data among these patients. Our findings indicate that there is a potentially higher level of liver damage in patients with HBV/HCV co-infection, warranting that those diagnosed with either HBV or HCV should also be tested for the other hepatitis virus. Screening of HBV infection can be made more widespread through molecular testing of HBV DNA by polymerase chain reaction to detect occult hepatitis B infection.

There were several limitations to our study. There were no available data on occult HBV infection since HBV DNA assays were not performed. This might underestimate the real burden of HBV in this study population. The HBV immunization status of patients was also unknown, which is something we can address in our future investigations. In this study, the majority of HBsAg and anti-HCV cases were initially diagnosed by clinical suspicion of an underlying disease, which is likely to underestimate the total number of viral hepatitis infections in our population, due to the presence of subclinical or asymptomatic infections. This study is limited to the review of medical records; no information was gathered directly from the patients.

Our results may be useful for making estimates and projections about future liver disease burden. A recently developed mathematical model by El-Bouzedi^30^, that uses available epidemiological data on viral hepatitis to estimate future consequences of hepatitis infection, can be incorporated in our future research. Such models will help to inform health policy, resource distribution, and healthcare delivery in India, as well as other countries. These types of studies can also lead to improved management of patients with viral hepatitis and inform health policy.

## Figures and Tables

**Table 1:** Demographic data on HBV/HCV distribution in the study population.

Demographic variables	HBsAg positive (%)	Anti-HCV positive (%)	HBV/HCV co-infection (%)	p-value*
Sex				
Male	1,364 (81.4)	201 (68.1)	22 (78.5)	p = 0.8
Female	310 (18.5)	94 (31.8)	6 (21.4)	

Age				
1–30	257 (15.35)	75 (25.4)	7 (25)	p = 0.001
31–60	754 (45.04)	105 (35.5)	13 (46.4)	
>60	663 (39.60)	115 (38.93)	8 (28.6)	

* Based on chi-squared test

**Table 2:** Liver function profile in HBV mono-infected patients and those with HBV/HCV co-infected patients.

Variables	HBsAg mono infection	HBV/HCV coinfection	p value*
ALT, mean±SD	114.4±403.8	100.25±201.4	0.001
ALT Abnormal high (n%)	156 (20.1%)	14 (50.0%)	
AST, mean±SD	94.7±344.8	106.3±265.7	0.001
AST Abnormal high (n%)	140 (18.1%)	8 (28.5%)	
ALP mean±SD	101.4 ± 71.6	114.7±65.9	0.001
ALP Abnormal high (n%)	132 (17.7%)	11 (39.2%)	
Bilirubin total mean±SD	1.4±2.9	1.7±2.7	0.001
Bilirubin total Abnormal high (n%)	151 (19.5%)	11 (39.2%)	
Bilirubin Direct mean±SD	0.7±2.7	1.05±2.7	0.001
Bilirubin Direct Abnormal high (n%)	368 (47.6%)	15 (53.3%)	
Bilirubin Indirect mean±SD	0.7±1.7	0.7±1.7	0.001
Bilirubin Indirect Abnormal high (n%)	110 (14.2%)	7 (25.0%)	

* Student’s t-test
